# From Conductive Hearing Loss to Whipple’s Surgery: A Case Report and Literature Review on Neuroendocrine Tumors of the Middle Ear

**DOI:** 10.7759/cureus.94654

**Published:** 2025-10-15

**Authors:** Lisa Schmitz, Mark Praetorius, Charlotte Kötting, Christian Stephan Betz, Tabita Breitsprecher

**Affiliations:** 1 Otolaryngology-Head and Neck Surgery, University Medical Center Hamburg-Eppendorf (UKE), Hamburg, DEU

**Keywords:** middle ear adenoma, middle ear neoplasms, middle ear neuroendocrine tumor, middle ear surgery, middle ear tumor, somatostatin receptor scintigraphy

## Abstract

Middle ear neuroendocrine tumors (MeNET) are a group of rare tumors and, therefore, challenging for ENT surgeons. Patients usually present nonspecific symptoms, and clinical and radiological examinations do not provide the sensitivity and specificity necessary to make a reliable diagnosis. Even intraoperatively, MeNET may be heterogeneous in its appearance. Consequently, a definitive diagnosis is challenging and based on a combined histopathology and immunohistochemical examination. These patterns, again, are wide-ranging. As a result, numerous terms have emerged in medical history, but they all describe very similar pathologies. Nowadays, we summarize them under MeNET. Overall, they show a rather less invasive growth behavior and appear mostly localized in the tympanic cavity. The number of reported cases of MeNET with highly invasive or metastatic growth is paltry. In this case report, we present a 65-year-old female patient with MeNET who complained of hearing loss and aural fullness in her left ear. After histological evaluation, a MeNET of pancreatic origin was assumed. Additional staging with DOTATOC showed an upregulated somatostatin expression in the pancreatic caput and cauda, and a suspected correlate in MRI was described, leading to a partial pancreatic resection. Surprisingly, histological analysis showed ultimately no evidence of NET in the pancreatic tissue after all.

## Introduction

Middle ear neuroendocrine tumors (MeNET), also previously referred to as middle ear adenoma [1], carcinoid of the middle ear [2], amphicrine adenomas of the middle ear [3,4], middle ear adenomatous neuroendocrine tumors (MEANTs) [5], or neuroendocrine adenoma of the middle ear (NAME) [6], are a rare and, admittedly, in at least some cases, an unexpected finding in exploratory tympanotomy. Since the visual appearances of MeNET under white light microscopy may vary [7,8], diagnosis can mostly only be made after histopathological and immunohistochemical analysis. Within the largest series of endocrinocarcinomas [9], in total 11,842, only 0.7% were assigned to localizations in the middle ear, testes, kidneys, or others [9]. In contrast, a much larger number of cases have been reported in the digestive tract (73.7%) and the bronchopulmonary system (25.1%) [9,10], resulting in a significant extension of availability for NET in those fields. Current guidelines, therefore, focus diagnosis and treatment predominantly on neuroendocrine neoplasms (NEN) of enteral, pancreatic, hepatic, or pneumological origin [11]. Thus, consensus about standard diagnostic and therapeutic procedures in cases of middle ear manifestations is pending. There is a lack of agreement about the histogenesis of middle ear neuroendocrine neoplasms because the reported lesions showed a high variety in architectural patterns and immunohistochemical markers [1]. Only a few cases reported showed a MeNET with extended disease with lymph nodular or distant metastasis [12-16], in all reported cases, the primary tumor was located in the middle ear, and metastases were osseous, cerebral, or hepatic. Herein, we present a case of a 65-year-old female patient diagnosed and treated with tympanic and suspected pancreatic neuroendocrine neoplasm.

## Case presentation

**Figure 1:**
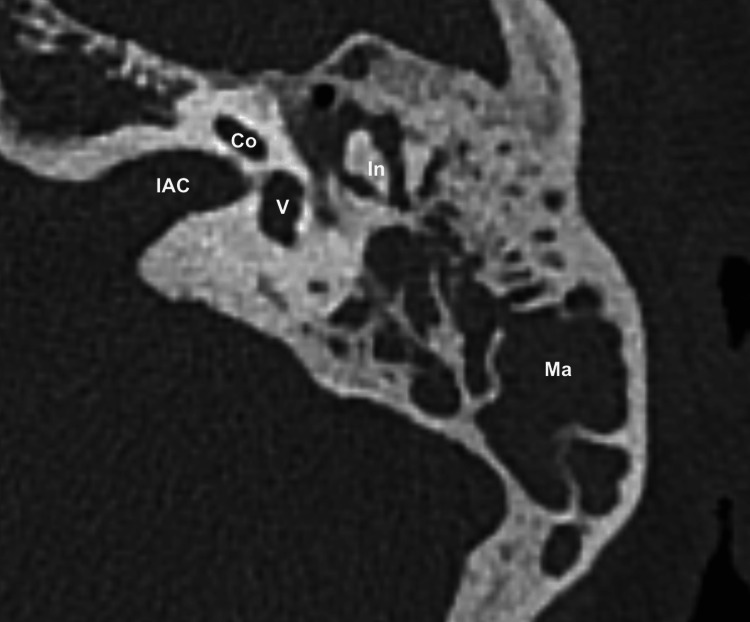
High-resolution CT of the left temporal bone showing an obstructed mastoid cavity and an obstructed tympanic cavity with no signs of erosion of the ossicles Co: Cochlea, V: Vestibule, In: Incus, IAC: Internal auditory canal, Ma: Mastoid

A 65-year-old woman consulted her ear, nose, and throat (ENT) specialist in a practice with left-sided hearing loss of unknown cause. Audiograms showed a mild mixed hearing loss and a type B tympanogram of the left ear. Examination showed a reduced transparency of the left tympanic membrane. No further abnormalities were noticed in the ENT examination. Therefore, the ENT specialist performed left paracentesis under local anesthesia because of suspected tympanic effusion, but no fluid was aspirable. Additionally, oral medication with antibiotics and corticosteroids was prescribed, which did not result in alleviation of symptoms. Consequently, high-resolution computed tomography of the temporal bone was initiated by the ENT specialist. Radiological findings described a left mastoid opacification without destruction of the mastoid cells and suspected left tympanic effusion without ossicular destruction. Therefore, radiographic findings were considered to be related to chronic mastoiditis (Figure 1).

The patient was then referred by the ENT practice to our department. During the first consultation at our outpatient department, the patient was still complaining about left aural fullness and hearing loss and reported for the first time about a left-sided tinnitus that had been present for years without affecting or disturbing her everyday life. Symptoms have been remarkably progressive over the past one to two years. Tympanic insufflation through Valsalva’s maneuver was reported to be possible without any side effects. Family medical history and occupational medical history showed no abnormalities. ENT-related surgeries were negated. Pre-existing illnesses were arterial hypertension, hypothyroidism, and hypercholesterolemia. The patient was on long-term medication with bisoprolol, hydrochlorothiazide, levothyroxine, simvastatin, and vitamins A and D, without any of these drugs being known to cause hearing loss. The left auricle and outer ear canal were inconspicuous. Microscopic examination showed a yellow mass behind the left tympanic membrane, but no signs of destruction. No signs of facial weakness could be detected. The tuning fork tests showed lateralization to the left in Weber’s test and a positive Rinne’s result for the right ear and a negative Rinne’s result for the left ear.

**Figure 2:**
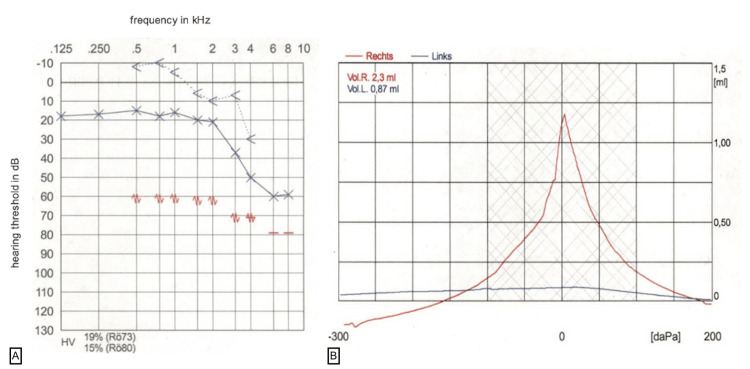
Left-sided pure-tone audiogram (A) and tympanogram (B) A: Showing a moderate combined hearing loss with PTA threshold for air conduction at 20 dB up to 2 kHz and an increase of the threshold up to 60 dB at 8-10 kHz. Maximum air-bone gap of 25 dB at 0.75 kHz. B: Showing a reduced tympanic membrane compliance left (blue line) and regular tympanic membrane compliance right (red line)

A pure tone audiogram showed normacusis for the right ear and for the left ear a mixed hearing loss with an air conduction hearing threshold of 20 dB from 0.125 to 2 kHz and an increase of the air conduction hearing threshold to 60 dB between 6 and 10 dB. The left bone conduction hearing threshold was between -10dB and 10dB from 0.125 to 3kHz, with an increase up to 30dB at 4kHz and no further derivable bone conduction threshold in higher frequencies. The maximum air-bone gap left was 30 dB at 3 kHz. The tympanogram showed reduced compliance on the left and normal compliance of the right tympanic membrane (Figure 2).

**Figure 3:**
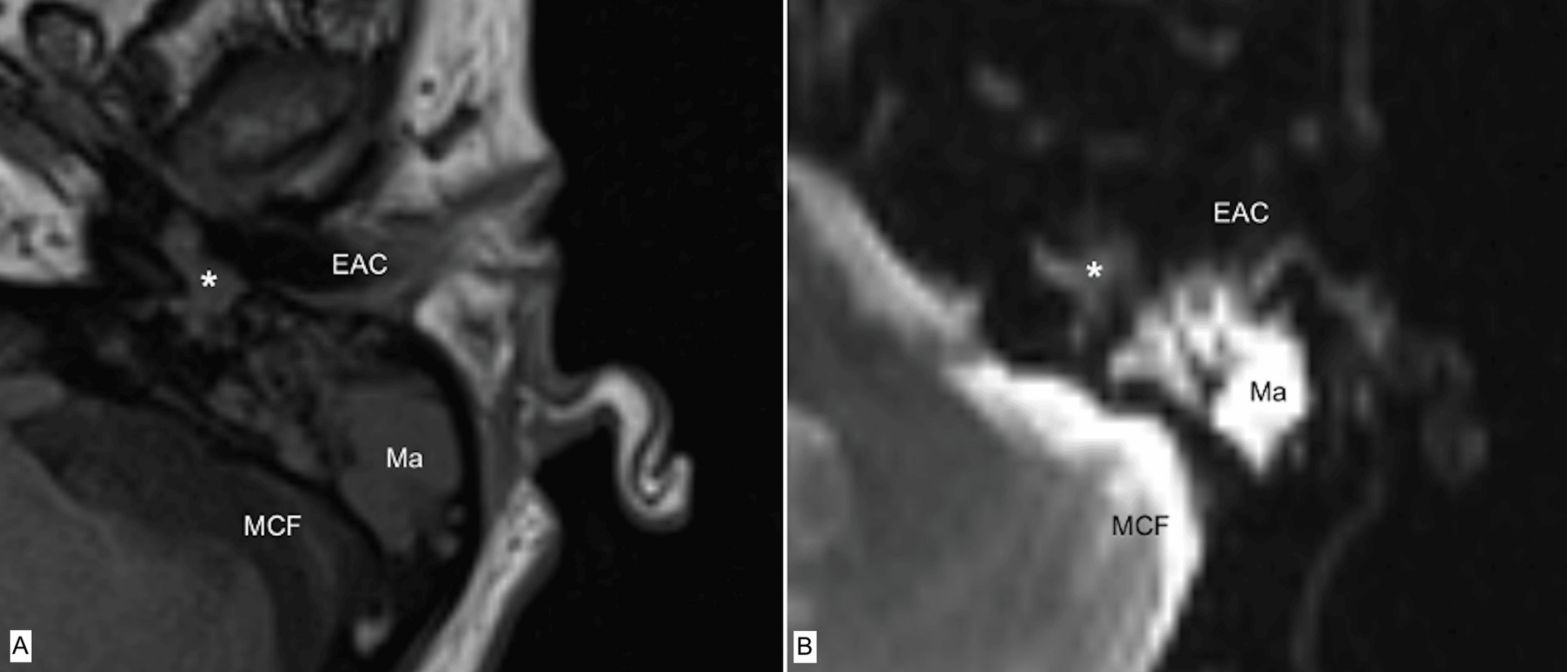
MRI of the left temporal bone showing a mass in the antrum (asterisk), later confirmed as MeNET A: T1-sequence, B: Diffusion-weighted sequence, Ma: Mastoid, EAC: External auditory canal, * indicating MeNET

Additional high-resolution MRI imaging with diffusion-weighted sequences of the temporal bone was requested in case of possible cholesteatoma. Herein, radiological findings showed a slightly diffusion-restrictive mass in DWI sequences and fluid restriction in the mastoid cells, believed to be in line with the diagnosis of cholesteatoma (Figure 3).

Based on the patient’s medical history and clinical and radiological examination, a tympanoplasty and optional mastoidectomy of the left side were indicated. Intraoperative findings showed a yellow mass wrapped around the ossicles without bony destruction and no signs of a typically located cholesteatoma. Therefore, a complete removal for histopathological examination could be successfully performed, initially maintaining the ossicular chain continuity. Mastoid opacification was intraoperatively assumed to be related to fluid restriction through the middle ear mass; therefore, no initial mastoidectomy but a mastoid biopsy was performed.

Clinical, pathological, and immunohistochemical analysis revealed a well-differentiated neuroendocrine tumor with positivity for AE1-AE3, synaptophysin, CK7, EMA, ISNM1, Islet-1, DPC4, and RB-1 and negativity for chromogranin A, CPA1, CK20, CDx2, S100, Sax10, p63, p40, CK5/6, c-KIT, DOG1, SMA, calcitonin, thyroglobulin, TTF-1, napsin, GATA-3, estrogen receptors, progesterone receptors, and Pax8. The Ki67 labeling index was less than 1%. More than 90% of the tumor cells showed a high nuclear positivity for SSTR2a. For better understanding, the findings of positive histological stains and their medical value are detailed in Table 1.

**Table 1:** Immunological stains and their medical value found in the patient's tumor tissue References 17-21

Stain	Description	Diagnostic use	Expression /Alteration
AE1/AE3	Monoclonal antibody cocktail to detect keratins	differentiation between carcinoma and non-epithelial tumors [17]	Adenocarcinoma, squamous Cell carcinoma, CUP, mesothelioma, thymic epithelial tumors
Synaptophysin	Membrane glycoprotein found in presynaptic vesicles	Differentiation of neoplasms of neural and non-neural origin [18]	Pheochromocytoma and paraganglioma (adrenal medulla), pancreatic islets, neuroblastoma, SCLC, medullary thyroid carcinoma
CK7	Cytokeratin 7 helps to maintain structural integrity and stability of epithelial cells	Differentiation between different types of epithelial tumors. Often used in combination with CK20 as CK7/CK 20 panel [19]	CK7+/CK20-: breast, lung, ovarian cancer CK7-/CK20+: colorectal and Merkel cell carcinomas CK7+/CK20+: upper GI-tract cancers, some pancreatic cancers CK7-/CK20-: prostate, hepatocellular carcinomas
EMA	Epithelial Membrane Antigen (also known as MUC1)	Intensity and pattern of EMA can provide information about aggressiveness and differentiation for tumors	Adenocarcinomas (various glandular tissues), Mesotheliomas, ovarian serous carcinomas, glioma, ependymoma, adenoid cystic carcinoma (salivary gland tumor)
INSM1	Insulinoma-associated protein 1	Diagnostic marker for neuroendocrine tumors [20]	Pancreas insulinoma, SCLC, Neuroendocrine tissues
Islet-1	Transcription factor protein playing an important role in embryonic development	ISL1-expression is upregulated in embryogenic tumors and the spectrum of neuroendocrine tumors [20,21]	Neuroblastoma, SCLC, prostate cancer (i.e., aggressive and metastatic disease), pancreatic cancer
DPC4 (Deleted in Pancreas Cancer Locus 4)	Tumor suppressor gene located on chromosome 18q21.1	Frequently mutated or deleted gene in various tumors, particularly pancreatic cancers. Alteration of DPC4 can lead to a dysfunction of TGF-beta signaling, contributing to tumor progression, invasion and metastasis	Pancreatic ductal adenocarcinoma, colorectal cancer, gastric cancer, cholangiocarcinoma, endometrial cancer
RB-1 (Retinoblastoma 1 gene)	Tumor suppressor gene located on chromosome 13q14	RB-1 inhibits progression from G1 phase to S-phase in the cell cycle, preventing excessive cell growth and division. Alterations lead to loss of tumor suppressor function and uncontrollable cell division.	Retinoblastoma, osteosarcoma, SCLC, bladder cancer, breast cancer, prostate cancer

**Figure 4:**
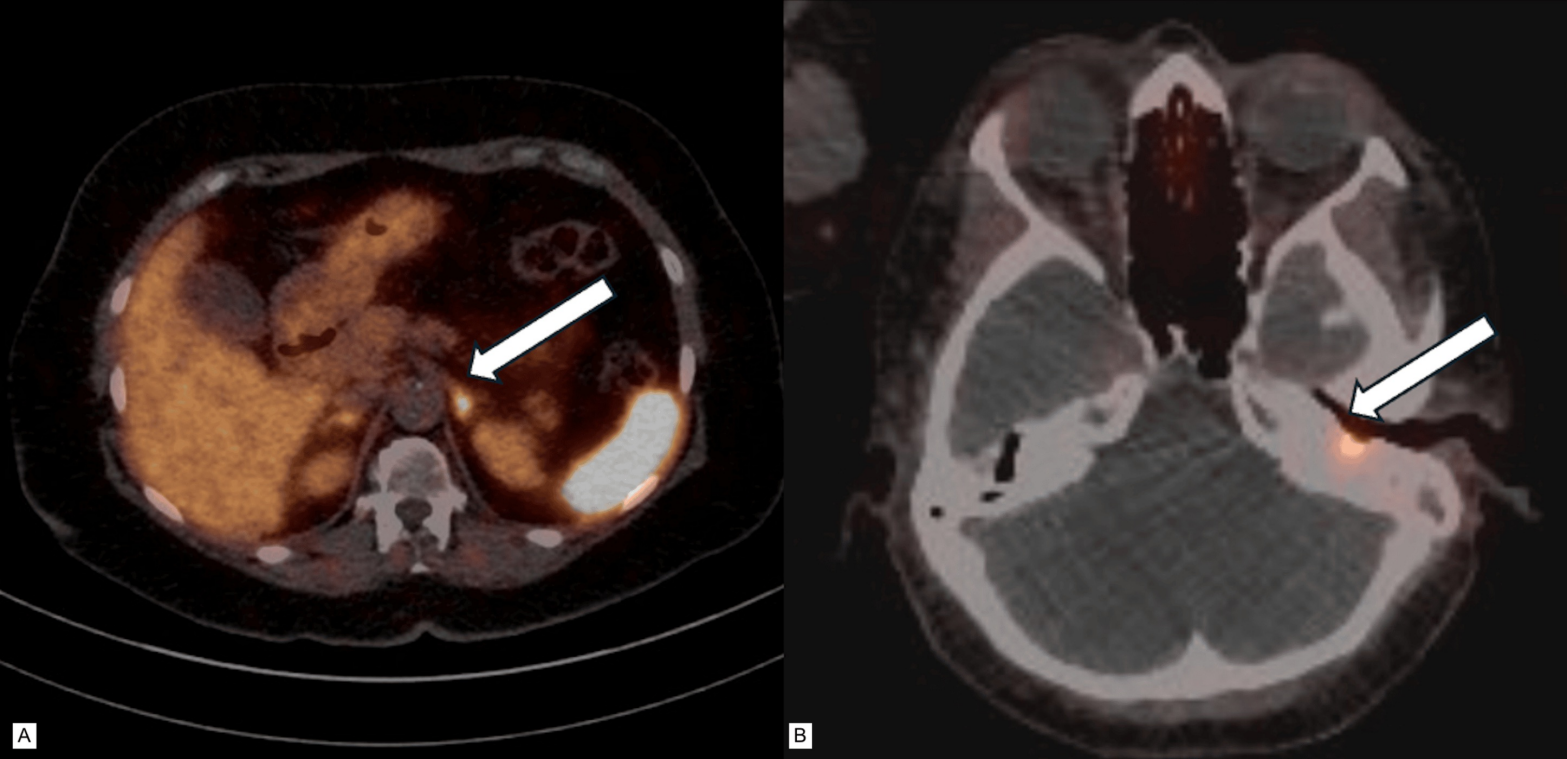
Selected areas of DOTAOC-enhancement from a full-body DOTATOC PET/CT scan in the diagnostic pathway for distant metastasis A: Abdomen: white arrow indicating DOTATOC enhancement of pancreatic corpus. B: Skull: white arrow indicating DOTATOC enhancement of the resection cavity in the middle ear

The case was presented in the tumor board for neuroendocrine pathologies. The consensus decision was the recommendation for a DOTATOC PET/CT scan for further staging. Imaging was performed and revealed a residual utilization of the tracer in the middle ear cavity, indicating a residual NET as well as tracer utilization in the pancreatic cauda (Figure 4).

**Figure 5:**
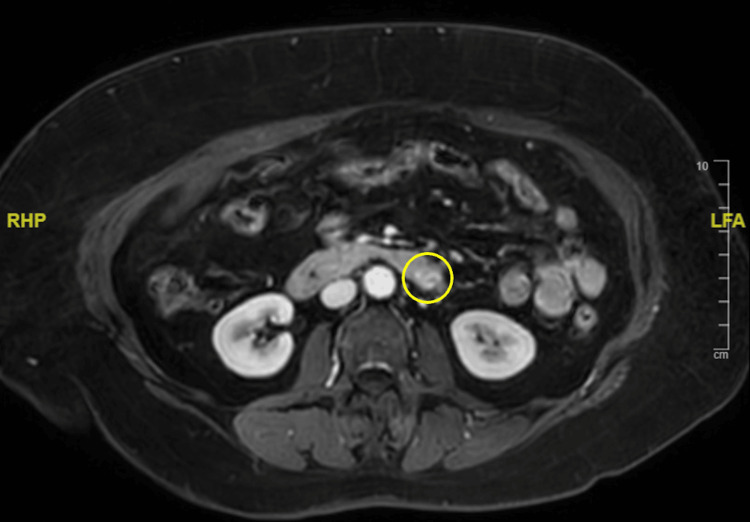
MRI scan (T2 weighted) of the abdomen Yellow circle: Outline of hypointense lesion of the pancreatic cauda

An MRI scan of the abdomen was performed to depict the pancreas in more detail (Figure 5).

In correlation to the preliminary PET/CT scan, a questionable hypointense lesion of the pancreatic cauda, which could be measured with certainty, was diagnosed. Again, the case was discussed in the tumor board for neuroendocrine pathologies. Due to a lack of cases with multilocular NEN of pancreatic origin, Whipple’s surgery was recommended.

Histopathological examination of the specimen from the partial pancreatectomy, however, did not show any solid tumor.

After prolonged rehabilitation due to abdominal wound dehiscence and two revision surgeries, a left tympano revision and mastoidectomy with partial removal of the ossicular chain were performed uneventfully six months after the visceral surgery to ensure complete resection. Since the incus appeared intact under microscopic examination, we decided to use an autologous incus interposition to prevent greater hearing loss. A partial ossicular replacement with a titanium prosthesis was avoided because of expected artifacts in follow-up imaging.

Histopathological examination of the mastoid and middle ear resection confirmed a small but completely removed residual tumor in the tympanic cavity. The mastoid margins showed no evidence of residual tumor. The postoperative tumor board discussed additional radiation therapy; however, due to complete resection and the absence of residual or metastatic disease, clinical and radiological follow-up, including CT scans and measurement of chromogranin A levels, was considered the most appropriate management. Owing to increased chromogranin A levels, systemic therapy with the somatostatin analogue lanreotide (Somatuline®) 120 mg (s.c.) every four weeks was initiated.

Since then, the patient underwent her first follow-up consultation three months later. CT scans of the temporal bone, thorax, and abdomen showed no signs of locoregional recurrence and stable levels of chromogranin A. Therefore, maintenance therapy with somatuline was decided upon.

The next follow-up is scheduled in six months and will include CT thorax and temporal bone, MRI abdomen, chromogranin A in serum, and 5HIES in the 24-hour urine.

## Discussion

Neuroendocrine tumors of the middle ear are a rare entity that can mimic a variety of symptoms, such as conductive hearing loss [22], aural fullness, otalgia [23], vertigo, facial nerve palsy, or otorrhea [24]. The histopathological patterns of MeNET are as variable as its symptomatic appearance. Torske et al. showed in a study of 48 cases that only nearly one-fifth of the tumors showed a single growth pattern [25]. Therefore, characterization and the resulting nomenclature are still matters of debate. In the 2022 update, the WHO classification of head and neck neuroendocrine neoplasms suggested the use of Middle Ear Neuroendocrine Tumor (MeNET) as a comprehensive term [26]. This term does not initially allow any conclusions to be drawn about the dignity or histological origin, but enables the creation of a larger pool of data for further research. MeNET includes both benign and malignant tumors with a high variance in clinical, histological, and metastasis patterns. Other than histopathological origins, there is a consensus on the treatment of choice regarding complete surgical resection. The majority of MeNET are confined in the mesotympanon; in cases with involvement of the ossicles, complete removal of the tumor mass and ossicles with reconstruction showed lower recurrence rates compared to ossicle-preserving surgery [25]. In general, prognosis is generally favorable with complete surgical removal, as MeNETs are slow-growing, rarely invasive, and less likely to metastasize in comparison to other malignancies. A retrospective study by van der Lans et al. Showed one out of nine cases with a locally invasive tumor growth [27], though surgical resection was still manageable following adjuvant radiotherapy.

Only a highly limited number of cases of neuroendocrine neoplasms with regional metastasis in lymphnodes [12,15,16] or distant metastasis [13,14] have been reported. Sun et al. recently reported a non-redactable primary MeNET with dural infiltration and multiple brain metastases [13]. Notably, cases reported with invasive growth or distant metastasis were, if analyzed and reported, more likely to show a Ki-67 labeling index over 3% [13,22,28]. As with other entities, the metastatic potential increases with higher grading. However, it is striking that in the case reports with NET confined to the middle ear, Ki67 was less than 3% when investigated and reported. Due to a lack of missing data of metastatic MeNET, a prognostic value of Ki67 can only be assumed, but Asa et al. summarized that locally invasive and metastatic disease showed higher Ki-67 labeling indices than confined disease [29]. Therefore, further research will be necessary to evaluate the diagnostic value of immunohistological analysis of MeNET. Despite immunological analysis, positron emission tomography/computed tomography (PET/CT) became part of the work-up of MeNET because no CT or MRI-specific findings are known to date for the primary tumor. Therefore, also, staging for metastasis is also based on an immunological feature of these entities. With the radiotracer 68Ga-DOTATOC PET/CT, somatostatin receptors (SSTRs), expressed in the majority of NET, can be targeted [30].

This is the first case describing localized and low-grade (G1) middle ear NET, which showed an overexpression of SSTR 2 in the pancreatic corpus and cauda in the 68Ga-DOTATOC PET/CT. Since a corresponding correlate was suspected in the supplementary MRI scan, an abdominal exploration was performed, and a partial pancreatic resection was carried out. Surprisingly, pancreatic NET could not be detected in the histopathological examination. However, to the best of our knowledge, no previously published case of a MeNET demonstrating SSTR2 expression in another organ has been reported where the SSTR2 expression did not lead to a distant tumor diagnosis. Therefore, we are able to report this case for the first time rather than compare it with existing literature.

Enhanced expression for SSTR2 might therefore be noticeable in endocrine-active tissue even without pathological value, but no valid data could be found in the literature. In the presented case, we have to admit that this patient retrospectively received an over-treatment with a comparatively high risk for morbidity due to a lack of supplementary tools in the diagnosis of MeNET. In cases of a low-grade and localized middle ear NET with distant abnormalities in PET/CT staging in the sense of suspected metastases, we therefore recommend a generous indication for further imaging and, if necessary, rechecking, as we have learned from this case that, in accordance with the currently available literature, distant metastasis is extremely unlikely in this constellation. Generally, we need to accrue more data on this rare entity to develop a valid diagnostic tree.

## Conclusions

This case underlines that neuroendocrine tumors of the middle ear are an entity that is challenging to diagnose. Literature showed metastatic potential and a high tendency for local recurrence; therefore, adequate treatment is essential. To date, complete surgical removal is suggested as the standard of therapy. We want to emphasize further research and reporting of cases to minimize potential pitfalls.
